# Mediterranean-Type Diet Adherence and Body Mass Index through 20 Years of Follow-Up: Results from the ATTICA Cohort Study (2002–2022)

**DOI:** 10.3390/nu16081128

**Published:** 2024-04-11

**Authors:** Evangelia Damigou, Michael Georgoulis, Christina Chrysohoou, Fotios Barkas, Elpiniki Vlachopoulou, Petros S. Adamidis, Evrydiki Kravvariti, Costas Tsioufis, Christos Pitsavos, Evangelos Liberopoulos, Petros P. Sfikakis, Demosthenes Panagiotakos

**Affiliations:** 1 Department of Nutrition and Dietetics, School of Health Sciences and Education, Harokopio University, 17676 Athens, Greece; edamigou@hua.gr (E.D.); mihalis.georgoulis@gmail.com (M.G.);; 2 First Cardiology Clinic, Medical School, National and Kapodistrian University of Athens, Hippokration Hospital, 15772 Athens, Greece; 3 Department of Internal Medicine, Medical School, University of Ioannina, 45500 Ioannina, Greece; 4 First Department of Propaedeutic Internal Medicine, Medical School, National and Kapodistrian University of Athens, Laiko General Hospital, 15772 Athens, Greece

**Keywords:** dietary habits, Mediterranean diet, obesity predisposition, obesity risk, weight trajectories, body mass index

## Abstract

Evidence of the association between dietary habits and long-term body weight status is scarce. This study aimed to evaluate changes in Mediterranean-type diet (MTD) adherence in relation to body weight during 20 years of follow-up. Data from n = 1582 participants from the ATTICA cohort study (2002–2022) were used. MTD adherence was assessed via MedDietScore, and body weight status via body mass index (BMI) by 3 different measurements. We found that MTD adherence and changes in this adherence were inversely related to BMI at 20 years and the mean BMI during the 20-year follow-up. In multi-adjusted linear regression models, a 1/55 increase in baseline, 10-year, and 20-year MedDietScore was associated with a decrease of 0.05–0.13 kg/m^2^ in BMI at 20 years and of 0.08–0.09 kg/m^2^ in the mean BMI. Being consistently close to the MTD for 20 years was associated with a >90% decreased risk of maintaining overweight/obesity during the 20-year period. Strong, protective, long-lasting effects of the MTD were observed, even in those who deviated from the MTD in the follow-up (41% of the sample). Our results highlight the need to focus on the overall diet quality to minimize the risk of maintaining an excessive body weight during the life-course.

## 1. Introduction

Obesity is a multifactorial chronic disease with significant health consequences, and is currently recognized as an epidemic. Body weight is a modifiable risk factor for the prevention of cardiometabolic disorders, some types of cancer, and other non-communicable diseases [1,2,3,4,5,6,7]. Based on a recent report from WHO, 6 out of 10 Europeans have a body weight on the overweight or obese scale, while approximately 2 out of 10 are on the obese scale [8]. Data from the Global Burden of Disease study have shown that in both sexes, the global burden of disease (i.e., global deaths and disability-adjusted life years-DALYs) attributed to high body mass index (BMI) was more than 2 times higher in 2017 compared to 1990 [9]. It was recently estimated that by 2035, globally, 3.3 billion adults will have overweight or obesity [10]. Moreover, it has been estimated that in the US alone, the obesity epidemic has cost USD 113.9 billion in direct healthcare costs [11].

The most widely acknowledged drivers of the pervasive obesity include an increased energy intake, decreased physical activity and low-quality diets, which, along with other factors, may lead to disruptions in an individual’s energy balance [12]. In a recent perspective paper, Mozaffarian [13] highlighted that, despite the increased obesity prevalence rates, national data from the US (where the highest obesity rates are observed) do not support increases in energy intake or decreases in physical activity. Thus, it is proposed that alternative factors that could be considered are biological mechanisms including metabolic expenditure, the gut microbiome, and the intergenerational transmission of risk, all of which are related to changes in diet quality [13]. 

Multiple dietary patterns have been studied in relation to the development of overweight/obesity or body weight management, the most studied being the Mediterranean dietary pattern [6,14,15,16,17,18]. However, studies exploring the association between adherence to the Mediterranean-type diet (MTD), especially longitudinal changes in MTD adherence, for which declines have been observed globally [19,20,21], and long-term body weight status, remain scarce.

Hence, our study aimed to evaluate MTD adherence patterns in relation to body weight during 20 years of follow-up. Specifically, we evaluated various MTD adherence trajectories in relation to body weight as assessed by (i) BMI at the 20-year follow-up (2022), (ii) mean BMI during the whole study period (2002–2022), (iii) BMI trajectories (during 2002–2022), and (iv) the risk of always having overweight/obesity during the 20-year follow-up. 

## 2. Materials and Methods

### 2.1. Study Design and Aims

The ATTICA study is a prospective cohort study, with 4 different evaluations; the baseline evaluation performed during 2001–2002, and 3 follow-up evaluations in 2006, 2012, and 2022 (i.e., 5-, 10- and 20-year follow-ups). The study’s main aim was to evaluate anthropometric, lifestyle, clinical, biochemical, and socio-demographic parameters related to cardiovascular disease (CVD) incidence. Adult men and women from the Attica region of Greece (including Athens, the capital of Greece) were included. Participants’ characteristics were evaluated by face-to-face interviews with the investigators. The study methodology and aims can be found in detail in other publications [22,23,24].

### 2.2. Bioethics

The ATTICA study was carried out in accordance with the Declaration of Helsinki (1989) of the World Medical Association and was approved by the Institutional Ethics committee of Athens Medical School (#017/1.5.2001). To participate in the study, all participants were informed about the study aims and provided written consent.

### 2.3. Baseline and Follow-Up Assessment

#### 2.3.1. Baseline Socio-Demographic Characteristics

Baseline socio-demographic parameters included age, sex, and socio-economic status (SES). High SES was defined as having 15 or more years of schooling and high income, or 10–14 years of schooling and very high income. Comparisons were made between high SES vs. all other SES categories (i.e., low/middle SES). 

#### 2.3.2. Baseline Cardiometabolic Parameters

Participants’ arterial blood pressure was measured with a manometric device (ELKA aneroid manometric sphygmometer, Von Schlieben Co., Munich, West Germany). Biochemical characteristics, i.e., glucose, insulin, total cholesterol, high-density lipoprotein cholesterol (HDL), triglycerides, high sensitivity C-reactive protein (hsCRP), uric acid, and creatinine, were measured in 12 h fasting blood samples using the chromatographic enzymic method in a Technicon automatic analyzer RA-1000 (Dade Behring, Marburg, Germany). Serum was harvested immediately after admission. Low-density lipoprotein cholesterol (LDL) was estimated by the Friedewald equation [25]. The homoeostasis model of assessment of insulin resistance (HOMA-IR) was used to estimate insulin resistance [26]. The estimated glomerular filtration rate (eGFR) was calculated through the Chronic Kidney Disease Epidemiology Collaboration equation as a measure of kidney function [27].

#### 2.3.3. Baseline Clinical Characteristics 

The presence of clinical characteristics was assessed by study investigators and defined according to WHO-ICD-10. Diabetes mellitus was defined as fasting plasma glucose ≥ 126 mg/dL or use of antidiabetic drugs [28]. Hypertension was defined as an average systolic blood pressure/diastolic blood pressure > 140/90 mmHg or use of antihypertensive drugs [29]. Hypercholesterolemia was defined as total cholesterol ≥ 200 mg/dL and/or use of hypocholesterolemic agents [30]. 

#### 2.3.4. Baseline Lifestyle Characteristics

Concerning smoking habits, participants were categorized as non-smokers (those who had never smoked) and as ever-smokers (those who currently or previously smoked). Pack-years of cigarette smoking were also calculated for each participant by multiplying smoking duration (in years) with the number of packs/day (assuming 20 cigarettes in a pack). 

To assess participants’ physical activity level, the short-form International Physical Activity Questionnaire (IPAQ), which is validated for the Greek population, was used [31]. The IPAQ has shown a very good reliability (i.e., r_w_ = 0.74) and validity (i.e., r_w_ = 0.72) in previous studies [32]. Comparisons were made between those who were inactive vs. those who were active (i.e., minimally/highly active, according to MET-minutes/week). 

#### 2.3.5. Dietary Habits

Participants’ dietary habits were evaluated by trained personnel through a Food Frequency Questionnaire (FFQ), validated for the Greek population, kindly provided by the developers [33]; this FFQ has a good overall reliability, as the assessment of most nutrients was comparable to 24 h recalls and the results were sufficiently reproducible after a 1-year period. Moreover, the overall dietary pattern of the participants was assessed through the level of adherence to the MTD. MTD adherence was evaluated via MedDietScore (range: 0–55, higher values signifying higher adherence) at baseline (2002) as well as at the 10-year and 20-year follow-ups (2012 and 2022, respectively) [34]. In a systematic review that assessed the psychometric properties of multiple Mediterranean diet scores, it was concluded that data on the applicability parameters and psychometric quality of these scores are scarce, but the MedDietScore, along with 2 other scores, had the highest levels of evidence [35]. MedDietScore is calculated based on 11 components, which include 7 Mediterranean foods/food groups traditionally consumed in the MTD (i.e., olive oil, fruits, vegetables, potatoes, legumes, whole grains, and fish), which are scored on a positive scale (0–5, for very rare to very frequent consumption); 3 non-Mediterranean foods/food groups (poultry, full-fat dairy products, and red meat), which are scored on the opposite scale; and alcohol, which is scored on a non-linear scale (0 for consumption of 0 and >7 servings/day, or 1 to 5 for consumption of 6–7, 5–6, 4–5, 3–4, and 1–3 servings/day, respectively). 

Furthermore, the following MTD trajectories were calculated during 2002–2012 and during 2002–2022, using the median value of MedDietScore at each time point (i.e., 27 at baseline, 26 at 10 years, and 22 at 20 years): always close (MedDietScore ≥ median both at baseline and the respective follow-up); from close to away (MedDietScore score ≥ median at baseline, but <median at the respective follow-up); from away to close (MedDietScore < median at baseline, but ≥median at the respective follow-up); and always away (MedDietScore < median both at baseline and the respective follow-up). 

#### 2.3.6. Body Weight Assessment 

Body weight and height were measured according to standard procedures at baseline (in 2002) as well as the 10- and 20-year follow-ups (in 2012 and 2022, respectively). BMI was computed as weight/height^2^ (kg/m^2^) at all evaluations. Moreover, the mean BMI of the 2002, 2012, and 2022 measurements was also calculated for each participant. Furthermore, body weight trajectories were calculated according to BMI measurements at baseline (in 2002) and at the 20-year follow-up (in 2022); participants’ body weight status was categorized as: (a) always had a normal weight: those who had a BMI < 25 kg/m^2^ at both follow-up evaluations (2002 and 2022); (b) acquired overweight/obesity: those who had a BMI < 25 kg/m^2^ in 2002, but ≥25 kg/m^2^ in 2022; (c) acquired a normal weight: those who had a BMI ≥ 25 kg/m^2^ in 2002, but <25 kg/m^2^ in 2022; and (d) always had overweight/obesity: those who had a BMI ≥ 25 kg/m^2^ at both evaluations (2002 and 2022). 

### 2.4. Study Sample

**Figure 1:**
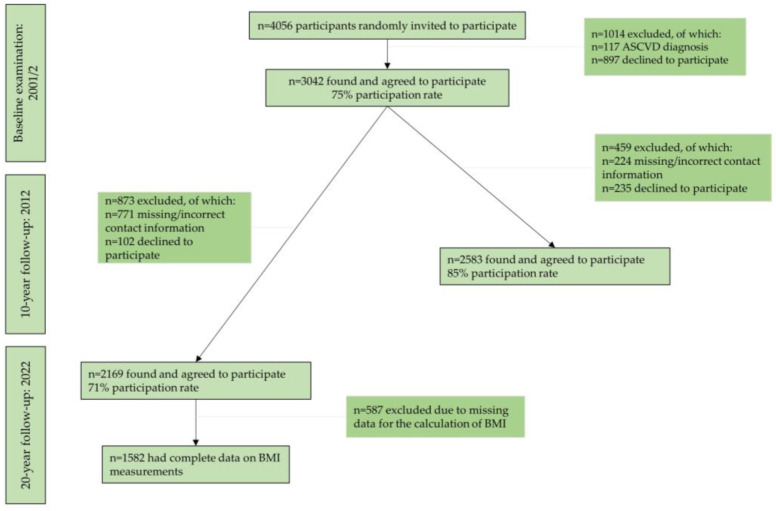
Flow-diagram of the ATTICA study participants (n = 1582). Abbreviations: ASCVD: atherosclerotic cardiovascular disease, BMI: body mass index.

During 2001–2002, 4056 adults from the region of Attica, Greece were randomly invited to participate in the study. The sample selection was random and stratified by age, sex, and region, based on the 2001 census. Initially, participants were reached by telephone calls, letters, or home visits. Afterwards, 3042 adult men and women, who suited the study criteria, agreed to participate in the baseline examination and were, thus, recruited in the study. Trained health professionals assessed these participants’ characteristics through face-to-face interviews. Of the 3042 initial participants, 2583 were found at the 10-year follow-up (85% participation rate), and 2169 participants were found at the 20-year follow-up (71% participation rate) (Figure 1). Almost half of the individuals lost to follow-up were not found because of missing or wrong addresses and telephone numbers and the rest because they declined to be re-examined. After excluding participants with missing values on the BMI measurements, the sample for the current analyses consisted of 1582 participants (Figure 1). No significant differences were observed between this sample and the baseline sample, regarding the distribution of age and sex (*p*-value > 0.05). 

### 2.5. Statistical Analysis

Categorical variables are presented as relative frequencies. Differences between body weight trajectories during 2002–2022 and categorical variables are derived from the chi-square test. Continuous variables are presented as mean values ± standard deviation (SD) for normally distributed variables (i.e., age) or as median (interquartile range-IQR) for non-normally distributed variables (i.e., triglycerides, HOMA-IR, hsCRP, eGFR, pack-years of cigarette smoking, and MedDietScore in 2002, 2012, and 2022). Continuous variables were tested for normality through probability–probability plots. Differences between body weight trajectories (2002–2022) and continuous variables were derived from one-way analysis of variance (ANOVA) for normally distributed variables or the Kruskal Wallis non-parametric test for non-normally distributed variables. *p*-values from post hoc comparisons were adjusted using the Bonferroni rule. Linear regression analysis was performed to evaluate dietary habits and BMI (in 2022 and during 2002–2022). All models were adjusted for age, sex, SES, energy intake, presence of hypercholesterolemia, hypertension, diabetes mellitus, physical activity level, and smoking status. Moreover, binary regression analysis was performed to evaluate dietary habits and the risk of longitudinally sustaining overweight/obesity during the 20-year follow-up. Crude models (Model 1) and adjusted models were used (Model 2: adjusted for age and sex; Model 3: Model 2 and further adjustment for SES, energy intake, smoking, physical activity, hypertension, hypercholesterolemia, and diabetes mellitus). Participants with missing values were excluded from the analysis. The final analyzed sample (n = 1582) was sufficient to obtain a statistical power of 80% or higher to evaluate two-sided statistical hypotheses of odds ratios of maintaining overweight/obesity of 1.20 or higher, with a 0.05 type-I error rate. For the statistical analyses, STATA version 17 (STATA Corp, College Station, TX, USA) was used. 

## 3. Results

In our study sample (n = 1582), mean BMI was 25.7 kg/m^2^ (SD: 4.5) at baseline, 26.6 kg/m^2^ (SD: 4.5) at 10 years, and 25.8 kg/m^2^ (SD: 4.6) at 20 years, while during the whole 20-year follow-up period, mean BMI was 26.0 kg/m^2^ (SD: 3.4). Moreover, trajectory analysis revealed that during the 20-year period, 49.4% of the participants (n = 781) always had overweight/obesity, 45.4% (n = 719) always had a normal weight, while 3.6% (n = 57) acquired overweight/obesity, and only 1.6% (n = 25) acquired a normal body weight. Concerning MTD trajectories, most of the study sample, i.e., 41% moved away, 32% were always away, 22% were always close and 5% moved closer to the MTD during the 20-year follow-up. 

### 3.1. Participants’ Baseline Characteristics by Body Weight Trajectories (2002–2022)

Table 1 presents participants’ baseline characteristics according to the different body weight trajectories during 2002–2022, as evaluated by BMI. In brief, compared to participants who always had a normal weight, those who always had a body weight in the overweight or obese range were older, more frequently men, exhibited a higher prevalence of all clinical factors (i.e., diabetes mellitus, hypercholesterolemia, and hypertension), had a more detrimental cardiometabolic profile (i.e., higher total and LDL cholesterol, triglycerides, blood pressure, HOMA-IR, and hsCRP levels, and lower HDL and e-GFR), reported higher pack-years of cigarette smoking, and were less physically active. Moreover, compared to those who always had a normal weight during 2002–2022, those who initially had an overweight/obese body weight in 2002 but had acquired a normal weight in 2022 were mainly older men who had a higher prevalence of hypercholesterolemia and hypertension, higher pack-years of cigarette smoking, higher levels of total and LDL cholesterol, higher levels of triglycerides, and higher blood pressure levels (Table 1).

### 3.2. Participants’ Dietary Habits by Body Weight Trajectories (2002–2022) 

Participants’ dietary habits according to body weight trajectories (2002–2022) are presented in Table 2. The overall dietary habits differed in the four body weight trajectories; participants in the categories “acquired a normal weight” and “always had overweight/obesity”, had lower MedDietScore values at baseline, compared to the other two categories (i.e., “always had a normal weight” and “acquired overweight/obesity”). Furthermore, participants in the categories “acquired a normal weight” and “always had overweight/obesity” also had lower MedDietScore values in 2012 and 2022, compared to participants who always had a normal weight. The majority of the participants showed declines in MTD adherence (high to low trajectory) during 2002–2012, while, during 2002–2022, a significant percentage were always away from the MTD (Table 2). 

### 3.3. Dietary Habits and Body Mass Index during the 20-Year Follow-Up (2002–2022)

Table 3 presents results from linear regression analysis, with dietary habits being the independent variable and the dependent variable being BMI in 2022 (for Model 1) and mean BMI during the whole 20-year period (2002–2022) (for Model 2). Inverse associations were observed between MedDietScore (at baseline, in 2012, or in 2022) and BMI in 2022 (Model 1, Table 3) or mean BMI during 2002–2022 (Model 2, Table 3), adjusted for age, sex, SES, energy intake, presence of hypercholesterolemia, hypertension, diabetes mellitus, physical activity level, and smoking status. A 1/55 increase in baseline MedDietScore was associated with a decrease of 0.13 kg/m^2^ in BMI at 20 years and of 0.08 kg/m^2^ in the mean BMI during 2002–2022; a 1/55 increase in MedDietScore in 2012 was associated with a decrease of 0.11 kg/m^2^ in BMI at 20 years and of 0.08 kg/m^2^ in the mean BMI; and a 1/55 increase in MedDietScore in 2022 was associated with a decrease of 0.05 kg/m^2^ in BMI at 20 years and of 0.09 kg/m^2^ in the mean BMI. Moreover, compared to being always away, being always or initially close to the MTD during the 10- or 20-year follow-up period was associated with decreases in BMI at 20 years or mean BMI during the 20 years ranging from 3.08 to 6.54 kg/m^2^ (for details please see Table 3). 

### 3.4. Dietary Habits and the Risk of Always Having Overweight/Obesity during the 20-Year Follow-Up (2002–2022)

Results from binary regression analysis to evaluate the relationship between dietary habits and the risk of always having overweight/obesity during the 20-year follow-up are presented in Table 4. In the fully adjusted model, adjusted for age, sex, SES, energy intake, smoking, physical activity, hypertension, hypercholesterolemia, and diabetes mellitus, adherence to the MTD, both at baseline and at the follow-ups, was inversely associated with the 20-year risk of persistently having overweight/obesity. Specifically, an increase of 1/55 in baseline MedDietScore (2002) was associated with an 8% decreased risk of always having overweight/obesity; an increase of 1/55 in 10-year MedDietScore (2012) was associated with a 7% decreased risk; and an increase of 1/55 in 20-year MedDietScore (2022) was associated with a 4% decreased risk. Compared to being always away, being initially or always close to the MTD during 2002–2012 was associated with 83% and 97% reduced risk of always having overweight/obesity during 2002–2012, respectively. Moreover, being initially or always close to the MTD during 2002–2022 was associated with 90% and 98% reduced risk of always having overweight/obesity during 2002–2022, respectively. 

## 4. Discussion

### 4.1. Main Findings

The present study aimed to assess dietary habits and their association with lifelong body weight status. We found that MTD adherence was inversely related to BMI at the 20-year follow-up and the mean BMI, which was based on three different measurements performed during the 20-year period. Additionally, adhering to the MTD was inversely associated with the risk of continuously having overweight/obesity during 20 years of follow-up, independently of age, sex, SES, energy intake, smoking status, physical activity, and presence of hypertension, hypercholesterolemia, and diabetes mellitus. Being consistently close to the MTD was associated with an astounding > 90% decreased risk of maintaining overweight/obesity during these 20 years of follow-up. Interestingly, strong protective long-lasting effects of the Mediterranean dietary pattern were observed, even in those who were initially close but had a declined MTD adherence at the follow-up (41% of the current total study sample). Our results highlight the need to focus on the overall diet quality to minimize the risk of maintaining an excessive weight during the life-course. 

Moreover, we observed that only a small percentage of the study sample significantly changed their body weight status during the 20-year follow-up (i.e., 5.2% changed their body weight status; 3.6% acquired overweight/obesity, and 1.6% acquired a normal body weight). However, these findings do not negate the fact that short-term variations in body weight could have taken place. In the short term, some individuals might successfully lose weight, but weight regain is common [36,37], and may ultimately lead to changes of little significance in the long-term, without proper modifications. In fact, in a review by Yannakoulia et al. [38], it was highlighted that diet modification is essential for weight loss and that dietary patterns such as the MTD or food-based diets may help in achieving weight loss. 

### 4.2. The Current State of the Research Field

Studies that evaluate changes in diet quality are scarce, but have observed similar findings [17,39,40,41,42]. For instance, in a study by Fung et al., in a combined cohort from the Nurses’ Health Study, the Health Professionals Follow-Up Study, and the Nurses’ Health Study II, it was found that an improvement in diet quality, evaluated by multiple scores (i.e., the Alternate Mediterranean Diet—aMED, the Alternate Healthy Eating Index 2010—AHEI2010, and the Dietary Approaches to Stop Hypertension adherence score—DASH) was associated with less weight gain over 4-year periods, especially in overweight participants [17]. These results are similar to those of our study; it is worth noting that in our study, participants’ BMI at all time points, and mean BMI, were slightly in the overweight range, while in the study by Fung et al., participants’ baseline BMI was in the normal range [17]. Taken together, the amelioration of diet quality is particularly important for those that have an increased body weight. Similarly, in the Multiethnic Cohort Study, changes in diet quality were assessed through four indexes i.e., aMED, HEI2015, AHEI2010, and DASH, and it was found that improvements in diet quality during a 10-year period were associated with smaller weight gain in most subgroup analyses performed (by race/ethnicity, baseline age, and BMI) [40]. However, it should be noted that these results support the idea that both consistently increasing diet quality and longitudinally improving diet quality are beneficial for body weight status. Our results support the idea that, concerning MTD adherence, the initial adherence has beneficial effects, but getting closer to the MTD later in life-course does not, after adjusting for major socio-economic, clinical, and lifestyle factors. In one of our previous reports from the ATTICA cohort study, we found that those who moved closer to the MTD were older participants, with deteriorated health statuses [43]. Thus, reverse causality could apply to them; improvements in (potentially long-established) dietary habits could have taken place due to medical advice to attenuate disease burden [43].

Our results are also in line with multiple guidelines and systematic reviews supporting the idea that adhering to the MTD has a prime role in managing and preventing obesity [3,6,15,17,44,45,46]. For instance, the most recent guidelines on medical nutrition therapy for managing overweight and obesity from the European Association for the Study of Obesity (EASO) and the European Federation of the Associations of Dietitians (EFAD) state that, among other dietary patterns, adherence to an MTD and diets that emphasize the consumption of fruits and vegetables, whole grains, pulses, nuts, and dairy foods, with or without calorie restriction, are vital for managing weight and ameliorating metabolic health [3]. A systematic review and meta-analysis of six prospective cohorts showed that being closer to the MTD was related to a 9% decreased risk of overweight/obesity and that, per 1-point increase in the Mediterranean diet score used, the risk of overweight/obesity was reduced by 2%, while a 1-point increase in the Mediterranean diet score was also related to 0.04 kg less weight gain [45]. It should be mentioned that our results shed light on the long-term relationship between (changes in) dietary habits and (changes in) body weight management, a topic that has not been adequately studied from a life-long perspective. 

Additionally, it is important to study the effect of the Mediterranean dietary pattern on weight control compared to other dietary patterns. A systematic review of five RCTs with ≥12 months of follow-up investigated the adherence to MTD, low-fat, low-carbohydrate, and American Diabetes Association diets [47]. It was shown that when compared to low-fat diets, the MTD produced greater weight loss, while when it was compared to the other two diets (i.e., the low-carbohydrate and the American Diabetes Association diets), no significant differences in weight loss were observed. Another systematic review of 16 RCTs on the relationship between central obesity and the MTD found that the MTD led to a reduction in waist circumference and visceral fat in 13/16 studies [48]. Of note, n = 7 studies were energy restricted; the beneficial effect of the MTD compared to the control group remained in 3 out of the 7 studies. In a cross-over RCT (n = 62 overweight adults), an MTD and a low-fat vegan diet were compared for a 16-week period, with no limitations on energy intake [49]. It was shown that a vegan diet and an MTD had comparable effects for weight loss in a short-term period (i.e., 16 weeks). However, it should be noted that when participants followed the low-fat vegan diet, but not the MTD, they reduced their energy intake. Concerning energy intake, an interesting comparison is the one between the results on obesity from the PREDIMED (Prevención con Dieta Mediterránea) and the PREDIMED-PLUS trials [15,50]. In the landmark PREDIMED study, the aim was to assess the effects of only the MTD and not other lifestyle factors; hence, no energy restriction was employed, but the MTD enriched with either nuts or extra virgin olive oil (EVOO) (compared to a lower fat diet) was related to minimal body weight changes and less gain in central adiposity [15,50]. In the ongoing PREDIMED-PLUS trial, MTD is accompanied by physical activity to attain energy restriction, and compared to an MTD alone; preliminary results propose that the intervention group (MTD and physical activity with an average energy restriction of about 100 kcal/day) outclasses the control group (only MTD) in terms of weight loss [15,51,52].

### 4.3. The Mediterranean-Type Diet and Body Weight Management: The Underlying Mechanisms

Traditionally, an MTD is based on minimally processed foods; it includes a high consumption of fats, particularly EVOO, low-glycemic index carbohydrates, wholegrain cereals, fruits, legumes, vegetables, nuts; a moderate consumption of fish, poultry, dairy, and red wine with meals; and a low consumption of red meat and sweets [15,53,54]. In a comprehensive review, Estruch and Ross describe in detail the impact of the MTD in obesity control and obesity-related diseases [15]. In brief, an MTD containing approximately 40% of energy from fat and 40% from carbohydrates leads to reductions in body weight and waist circumference when energy restricted, and has beneficial effects for CVD and cardiometabolic factors (such as blood pressure, diabetes, hypercholesterolemia, and inflammation), as well as some types of cancer, neurodegenerative disorders, and mortality from all causes [15]. 

Recently, Dominguez et al. reviewed the role of the MTD to prevent and tackle obesity, as well as the molecular mechanisms that might explain this relationship [6]. Specifically, the components of the Mediterranean dietary pattern that may help in preventing obesity and managing body weight include the high content of polyphenols in EVOO, fruits, vegetables, herbs, spices, whole grains, legumes, and nuts; the high content of monounsaturated fatty acids (MUFAs) and poly-unsaturated-to-saturated fatty acid ratios (PUFA/SFA) due to the consumption of EVOO, nuts, fish, and seafood; and the high ratios of *n*-3/*n*-6 fatty acids due to nut, fish, and seafood consumption. All of these components have anti-inflammatory and anti-oxidant abilities that can help in reducing the chronic inflammation that is associated with obesity [6,55]. Moreover, vegetables and fruits, whole grains, legumes, and nuts are rich in fiber, which may help in achieving better appetite control, due to a lower gastric emptying and an increased perceived satiety [56]. Furthermore, polyphenols, *n*-3 PUFA, and fiber have prebiotic abilities and may be beneficial for the altered composition of the gut microbiota observed in patients with obesity compared to those with normal body weight [57,58]. Finally, a potential explanation for the protective nature of the MTD against the risk of having an increased BMI could be summarized by Aristotle’s quote “The whole is greater than the sum of its parts”. That is to say, the combination of different foods in a diet, and by extent the different nutrients in the food matrix, can act together and synergistically and multi-directionally affect disease risk [59], as well as body weight as observed in the current study. 

It should be noted that despite the high energy content of olive oil, it has been shown in the SUN (Seguimiento Universidad de Navarra) study that high consumption of olive oil, in the context of the MTD, was not associated with gaining weight or a higher risk of overweight/obesity [60]. The high calorie content of a specific food does not necessarily lead to an energy imbalance when the overall diet is taken into account. In addition, a systematic review and meta-analysis of randomized controlled trials (RCTs) concluded that a diet enriched with olive oil was beneficial for weight management in adults free of CVD [61], a population similar to that of our study sample. 

It has been shown that decreasing energy balance by even 50–100 kcal/day is attainable and can help in achieving weight loss [62]. Multiple diets can lead to weight loss and help in preventing and managing obesity if energy restricted, at least in the short term; as the dogma of thermodynamic laws states, “a calorie is a calorie” [16]. However, it might not be as simple as that, from a long-term perspective. It has been questioned if all calories are created equal. That is to say, different foods and nutrients are digested differently and can affect both energy intake and output, and by extent, energy balance; in the long-term, even a small, virtually unnoticed change in diet quality can be associated with gaining weight [63,64]. Herein, we found that increased diet quality, and specifically, higher MTD adherence, can offer improvements for body weight in the long term, independently of energy intake and other confounding factors. Recently, in a systematic review by Koutras et al. [65], it was found that a healthy dietary pattern, similar to an MTD (i.e., high intake of whole grains, fruits and vegetables, unprocessed cereals, and dairy, and low intake of sweets and high-fat foods), was related to longitudinal changes in body weight status (assessed as body weight or BMI) and overweight/obesity risk, which is in line with our results. 

### 4.4. Implications and Suggestions for Policy Measures 

According to the recently published World Obesity Atlas 2024 [10], the global cost associated with high BMI will exceed USD 4 trillion in the next 10 years (i.e., in 2035). However, investments to manage the pervasive obesity need to be made to reduce this cost [10]. Facilitating adherence to Mediterranean or other similar dietary patterns might be a relatively cost-effective measure to prevent the maintenance of increased adiposity, as shown in our study. Specifically, our results suggest that an initial or continuous increased diet quality, through an MTD, should be promoted. By extension, the focus should be shifted from counting calories to increasing the consumption of nutritious foods and following healthy diets [63], which, ultimately, might be more effective in achieving a negative energy balance. Moreover, this should be done as early as possible. That is to say, programs for obesity prevention and treatment should be implemented in schools or the community, and promote first and foremost a diet of high quality such as the MTD, along with other important lifestyle habits (e.g., increased physical activity, adequate sleep, and decreased television watching) [63]. This study also suggests that in public health actions, the focus could also be turned to preventing (further) weight gain rather than finding new ways (or fad diets) to induce weight loss [63]. For all these to be achieved, there needs to be a collaboration between the scientific community and the food industry, along with governmental authorities for the development and provision of healthier foods, which can then be easily incorporated in a person’s daily diet [64]. 

### 4.5. Strengths and Limitations

The ATTICA cohort study is a prospective cohort study with multiple follow-ups and a large sample, studied for 20 years (2002–2022), and therefore allowed the comprehensive investigation of the association between dietary habits and body weight over a long-term period. Moreover, the outcome variable was studied in multiple ways; BMI was studied at 20 years after the initial examination, as well as during the whole 20-year period (i.e., as BMI trajectories or as the mean BMI through three different examinations). Furthermore, MedDietScore was also evaluated at all three time points (in 2002, 2012, 2022); hence, changes in dietary habits were also assessed through MTD adherence.

Some limitations should also be acknowledged. Dietary habits were studied with an FFQ, validated for the Greek population and administered by trained personnel; however, there still exists the possibility of some measurement error attributed to the misreporting of dietary habits. Moreover, BMI does not distinguish between fat and fat-free mass; however, BMI is the most commonly used measure to screen for overweight or obesity in the majority of epidemiological studies [66]. Lastly, even though adjustments were performed for all the major socio-demographic, lifestyle, and clinical factors, some residual confounding due to other factors, such as sleep habits or psychological characteristics, might remain. 

## 5. Conclusions

This study found that following the Mediterranean dietary pattern can offer benefits for managing long-term body weight, as assessed by BMI, and also protect against the risk of persistently having overweight/obesity during a 20-year period of follow-up. While the strongest effects were observed in those who continuously adhered to the MTD, protective effects were also observed, even in those who were initially close but had a declined MTD adherence in the follow-up. Hence, the study results further strengthen the literature that emphasizes the protective effects of the MTD, by highlighting the long-lasting effects of this widely acknowledged dietary pattern to effectively manage body weight in the long term. To regulate the pervasive obesity, the focus should be shifted to diet quality, and collaborative efforts between the food industry, governmental authorities, and scientists should be focused on developing and providing consumers with healthier food choices. 

## Figures and Tables

**Table 1:** Participants’ baseline characteristics by body weight trajectories (2002–2022) in the ATTICA study (n = 1582).

	Trajectories of Body Weight, 2002–2022				*p*-Value *
Socio-Demographic Characteristics	Always Had a Normal Weight	Acquired Overweight/Obesity	Acquired a Normal Weight	Always Had Overweight/Obesity	
**N**	719	57	25	781	-
Age, mean (SD)	36 (10)	38 (10)	49 (10) ^a,b^	40 (9) ^a^	<0.001
Sex, %men	31	30	76 ^a,b^	65 ^a,b^	<0.001
High socio-economic status, %	39	32	41	38	0.784
**Clinical characteristics**					
Diabetes mellitus, %	1.3	3.7	0	5 ^a^	<0.001
Hypercholesterolemia, %	24	30	56 ^a^	40 ^a^	<0.001
Hypertension, %	11	20	43 ^a^	32 ^a^	<0.001
**Cardiometabolic** **parameters**					
Total cholesterol, mg/dL, mean (SD)	178 (37)	182 (43)	214 (43) ^a,b^	193 (40) ^a^	<0.001
LDL, mg/dL, mean (SD)	109 (34)	117 (40)	137 (42) ^a^	123 (37) ^a^	<0.001
HDL, mg/dL, mean (SD)	53 (14)	49 (12)	47 (16)	45 (13) ^a^	<0.001
Triglycerides, mg/dL, median (IQR)	70 (46)	79 (73)	105 (65) ^a^	107 (82) ^a,b^	<0.001
SBP, mmHg, mean (SD)	112 (15)	119 (17) ^a^	129 (20) ^a^	123 (16) ^a^	<0.001
DBP, mmHg, mean (SD)	73 (10)	77 (11)	85 (12) ^a,b^	81 (11) ^a,b^	<0.001
HOMA-IR, median (IQR)	2.6 (0.9)	2.6 (0.8)	2.8 (1.0)	2.9 (1.1) ^a^	<0.001
hsCRP, mg/L, median (IQR)	0.60 (1.3)	0.62 (1.1)	1.5 (2.0)	1.4 (2.1) ^a,b^	<0.001
eGFR, mL/min/1.73 m^2^, median (IQR)	142 (112)	146 (113)	129 (120)	126 (113) ^a^	0.021
**Lifestyle characteristics**					
Ever smokers, %	54	59	52	60	0.072
Pack-years of cigarette smoking, median (IQR)	280 (420)	260 (440)	433 (590) ^a,b^	420 (600) ^a^	<0.001
Physically active, %	40	33	36	31 ^a^	0.009

* *p*-values referring to differences between body weight trajectories and categorical variables: derived from the chi-square test. *p*-values referring to differences between body weight trajectories and continuous variables: derived from one-way ANOVA (i.e., age, normally distributed) or the Kruskal Wallis test (triglycerides, HOMA-IR, hsCRP, eGFR, pack-years of cigarette smoking: not normally distributed). ^a^ *p* < 0.05: *p*-values from post hoc comparisons (vs. always had a normal weight 2002–2022), adjusted using the Bonferroni rule. ^b^ *p* < 0.05: *p*-values from post hoc comparisons (vs. acquired overweight/obesity 2002–2022), adjusted using the Bonferroni rule. Abbreviations: ANOVA: analysis of variance; hsCRP: high-sensitivity C-reactive protein; DBP: diastolic blood pressure; eGFR: estimated glomerular filtration rate; IQR: interquartile range; HDL: high-density lipoprotein cholesterol; HOMA-IR: homeostatic model of assessment for insulin resistance; LDL: low-density lipoprotein cholesterol; SBP: systolic blood pressure; SD: standard deviation.

**Table 2:** Participants’ dietary habits by body weight trajectories (2002–2022) in the ATTICA study (n = 1582).

	Trajectories of Body Weight, 2002–2022				*p*-Value *
Dietary Habits	Always Had a Normal Weight	Acquired Overweight/Obesity	Acquired a Normal Weight	Always Had Overweight/Obesity	
N	719	57	25	781	-
Baseline MedDietScore (2002), median (IQR)	28 (2.3)	28 (2.1)	26 (2.3) ^a,b^	25 (2.7) ^a,b^	<0.001
MedDietScore (2012), median (IQR)	27 (2.4)	27 (2.2)	25 (2.6) ^a^	25 (2.8) ^a,b^	<0.001
MedDietScore (2022), median (IQR)	22 (4.0)	22 (6.0)	20 (4) ^a^	19 (3.0) ^a^	<0.001
Mediterranean-type diet trajectories 2002–2012, %			^a,b^	^a,b^	<0.001
Always low	8	2	72	56	
Low to high	1	2	12	6	
High to low	2	8	0	2	
Always high	89	88	16	36	
Mediterranean-type diet trajectories 2002–2022, %			^a,b^	^a,b^	<0.001
Always low	7	4	64	54	
Low to high	2	0	20	8	
High to low	45	55	12	31	
Always high	46	41	4	7	

* *p*-values referring to differences between body weight trajectories and categorical variables: derived from the chi-square test. *p*-values referring to differences between body weight trajectories and continuous variables: derived from the Kruskal Wallis non-parametric test (i.e., MedDietScore in 2002, 2012, 2022: not normally distributed). Bold indicates statistical significance. ^a^ *p* < 0.05: *p*-values from post hoc comparisons (vs. always had a normal weight 2002–2022), adjusted using the Bonferroni rule. ^b^ *p* < 0.05: *p*-values from post hoc comparisons (vs. acquired overweight/obesity 2002–2022), adjusted using the Bonferroni rule. Abbreviations: IQR: interquartile range.

**Table 3:** Results from linear regression analysis evaluating dietary habits and body mass index in the ATTICA study (n = 1582).

Dietary Habits (Independent Variables)	Models *			
	Model 1: BMI in 2022 (kg/m^2^), b (SE)	*p*-Value	Model 2: Mean BMI during 2002–2022 (kg/m^2^), b (SE)	*p*-Value
Baseline MedDietScore (2002), per 1/55	−0.13 (0.02)	<0.001	−0.08 (0.02)	<0.001
MedDietScore (2012), per 1/55	−0.11 (0.02)	<0.001	−0.08 (0.02)	<0.001
MedDietScore (2022), per 1/55	−0.05 (0.02)	0.019	−0.09 (0.02)	<0.001
Mediterranean-type diet trajectories 2002–2012 vs always low				
Low to high	0.30 (0.73)	0.683	−0.26 (0.55)	0.639
High to low	−4.09 (0.77)	<0.001	−3.08 (0.60)	<0.001
Always high	−4.37 (0.48)	<0.001	−3.37 (0.38)	<0.001
Mediterranean-type diet trajectories 2002–2022 vs always low				
Low to high	−0.47 (0.64)	0.459	−0.81 (0.57)	0.159
High to low	−3.88 (0.43)	<0.001	−3.16 (0.35)	<0.001
Always high	−6.54 (0.54)	<0.001	−5.28 (0.49)	<0.001

* Each line represents the dietary habits (dependent variables). The numbers in each cell represent the coefficients (b-coefficient and SE) of BMI in different time points (the independent variables) as indicated in each column. All models were adjusted for age, sex, socio-economic status, energy intake, hypercholesterolemia, hypertension, diabetes mellitus, physical activity, and smoking. Abbreviations: BMI: body mass index; SE: standard error.

**Table 4:** Results from binary regression analysis evaluating dietary habits and the risk of always having overweight/obesity during 20 years of follow-up in the ATTICA study sample (n = 1582).

Dietary Habits (Independent Variables)	Models		
	Model 1: Crude	Model 2: Age- and Sex-Adjusted	Model 3: Adjusted for Age, Sex, SES, Energy Intake, Smoking, Physical Activity, Hypertension, Hypercholesterolemia and Diabetes Mellitus
	OR (95%CI) *	OR (95%CI) *	OR (95%CI) *
Baseline MedDietScore (2002), per 1/55	0.81 (0.78, 0.85)	0.88 (0.86, 0.92)	0.92 (0.89, 0.95)
MedDietScore (2012), per 1/55	0.84 (0.81, 0.87)	0.90 (0.88, 0.93)	0.93 (0.91, 0.96)
MedDietScore (2022), per 1/55	0.93 (0.92, 0.95)	0.96 (0.94, 0.97)	0.96 (0.94, 0.99)
Mediterranean-type diet trajectories 2002–2012 vs always low			
Low to high	0.84 (0.40, 1.74)	0.83 (0.40, 1.72)	1.28 (0.44, 3.65)
High to low	0.11 (0.05, 0.24)	0.13 (0.06, 0.28)	0.17 (0.07, 0.42)
Always high	0.07 (0.05, 0.09)	0.08 (0.05, 0.11)	0.03 (0.03, 0.12)
Mediterranean-type diet trajectories 2002–2022 vs always low			
Low to high	0.47 (0.27, 0.84)	0.57 (0.32, 1.03)	0.70 (0.29, 1.67)
High to low	0.11 (0.08, 0.15)	0.08 (0.06, 0.12)	0.10 (0.05, 0.17)
Always high	0.02 (0.01, 0.03)	0.01 (0.01, 0.02)	0.02 (0.01, 0.05)

* OR and 95%CI concern the risk of always having overweight/obesity during the 20-year follow-up (2002–2022), compared to all other body weight trajectories (i.e., always having a normal weight, acquiring a normal weight, acquiring overweight/obesity). Abbreviations: 95%CI: 95% confidence interval; OR: odds ratio.

## Data Availability

The data presented in this study are available on request from the corresponding author. The data are not publicly available due to privacy restrictions.

## References

[1] Visseren F.L.J., Mach F., Smulders Y.M., Carballo D., Koskinas K.C., Bäck M., Benetos A., Biffi A., Boavida J.-M., Capodanno D. (2021). 2021 ESC Guidelines on Cardiovascular Disease Prevention in Clinical Practice. Eur. Heart J..

[2] Magnussen C., Ojeda F.M., Leong D.P., Alegre-Diaz J., Amouyel P., Aviles-Santa L., De Bacquer D., Ballantyne C.M., Bernabé-Ortiz A., Global Cardiovascular Risk Consortium (2023). Global Effect of Modifiable Risk Factors on Cardiovascular Disease and Mortality. N. Engl. J. Med..

[3] Hassapidou M., Vlassopoulos A., Kalliostra M., Govers E., Mulrooney H., Ells L., Salas X.R., Muscogiuri G., Darleska T.H., Busetto L. (2023). European Association for the Study of Obesity Position Statement on Medical Nutrition Therapy for the Management of Overweight and Obesity in Adults Developed in Collaboration with the European Federation of the Associations of Dietitians. Obes. Facts.

[4] Pati S., Irfan W., Jameel A., Ahmed S., Shahid R.K. (2023). Obesity and Cancer: A Current Overview of Epidemiology, Pathogenesis, Outcomes, and Management. Cancers.

[5] Boutari C., Mantzoros C.S. (2022). A 2022 Update on the Epidemiology of Obesity and a Call to Action: As Its Twin COVID-19 Pandemic Appears to Be Receding, the Obesity and Dysmetabolism Pandemic Continues to Rage On. Metabolism.

[6] Dominguez L.J., Veronese N., Di Bella G., Cusumano C., Parisi A., Tagliaferri F., Ciriminna S., Barbagallo M. (2023). Mediterranean Diet in the Management and Prevention of Obesity. Exp. Gerontol..

[7] Godos J., Zappalà G., Bernardini S., Giambini I., Bes-Rastrollo M., Martinez-Gonzalez M. (2017). Adherence to the Mediterranean Diet Is Inversely Associated with Metabolic Syndrome Occurrence: A Meta-Analysis of Observational Studies. Int. J. Food Sci. Nutr..

[8] World Health Organization WHO European Regional Obesity Report 2022. https://www.who.int/europe/publications/i/item/9789289057738.

[9] Dai H., Alsalhe T.A., Chalghaf N., Riccò M., Bragazzi N.L., Wu J. (2020). The Global Burden of Disease Attributable to High Body Mass Index in 195 Countries and Territories, 1990-2017: An Analysis of the Global Burden of Disease Study. PLoS Med..

[10] World Obesity Day Atlases|Obesity Atlas 2024. https://data.worldobesity.org/publications/?cat=22.

[11] Tsai A.G., Williamson D.F., Glick H.A. (2011). Direct Medical Cost of Overweight and Obesity in the USA: A Quantitative Systematic Review. Obes. Rev..

[12] Hill J.O., Wyatt H.R., Peters J.C. (2012). Energy Balance and Obesity. Circulation.

[13] Mozaffarian D. (2022). Perspective: Obesity-an Unexplained Epidemic. Am. J. Clin. Nutr..

[14] Poulimeneas D., Anastasiou C.A., Santos I., Hill J.O., Panagiotakos D.B., Yannakoulia M. (2020). Exploring the Relationship between the Mediterranean Diet and Weight Loss Maintenance: The MedWeight Study. Br. J. Nutr..

[15] Estruch R., Ros E. (2020). The Role of the Mediterranean Diet on Weight Loss and Obesity-Related Diseases. Rev. Endocr. Metab. Disord..

[16] Thom G., Lean M. (2017). Is There an Optimal Diet for Weight Management and Metabolic Health?. Gastroenterology.

[17] Fung T.T., Pan A., Hou T., Chiuve S.E., Tobias D.K., Mozaffarian D., Willett W.C., Hu F.B. (2015). Long-Term Change in Diet Quality Is Associated with Body Weight Change in Men and Women123. J. Nutr..

[18] Pavlidou E., Papadopoulou S.K., Fasoulas A., Papaliagkas V., Alexatou O., Chatzidimitriou M., Mentzelou M., Giaginis C. (2024). Diabesity and Dietary Interventions: Evaluating the Impact of Mediterranean Diet and Other Types of Diets on Obesity and Type 2 Diabetes Management. Nutrients.

[19] Damigou E., Faka A., Kouvari M., Anastasiou C., Kosti R.I., Chalkias C., Panagiotakos D. (2023). Adherence to a Mediterranean Type of Diet in the World: A Geographical Analysis Based on a Systematic Review of 57 Studies with 1,125,560 Participants. Int. J. Food Sci. Nutr..

[20] Vilarnau C., Stracker D.M., Funtikov A., da Silva R., Estruch R., Bach-Faig A. (2019). Worldwide Adherence to Mediterranean Diet between 1960 and 2011. Eur. J. Clin. Nutr..

[21] da Silva R., Bach-Faig A., Raidó Quintana B., Buckland G., Vaz de Almeida M.D., Serra-Majem L. (2009). Worldwide Variation of Adherence to the Mediterranean Diet, in 1961–1965 and 2000–2003. Public Health Nutr..

[22] Pitsavos C., Panagiotakos D.B., Chrysohoou C., Stefanadis C. (2003). Epidemiology of Cardiovascular Risk Factors in Greece: Aims, Design and Baseline Characteristics of the ATTICA Study. BMC Public Health.

[23] Panagiotakos D.B., Georgousopoulou E.N., Pitsavos C., Chrysohoou C., Metaxa V., Georgiopoulos G.A., Kalogeropoulou K., Tousoulis D., Stefanadis C., ATTICA Study group (2015). Ten-Year (2002–2012) Cardiovascular Disease Incidence and All-Cause Mortality, in Urban Greek Population: The ATTICA Study. Int. J. Cardiol..

[24] Damigou E., Kouvari M., Chrysohoou C., Barkas F., Kravvariti E., Pitsavos C., Skoumas J., Michelis E., Liberopoulos E., Tsioufis C. (2023). Lifestyle Trajectories Are Associated with Incidence of Cardiovascular Disease: Highlights from the ATTICA Epidemiological Cohort Study (2002–2022). Life.

[25] Friedewald W.T., Levy R.I., Fredrickson D.S. (1972). Estimation of the Concentration of Low-Density Lipoprotein Cholesterol in Plasma, without Use of the Preparative Ultracentrifuge. Clin. Chem..

[26] Matthews D.R., Hosker J.P., Rudenski A.S., Naylor B.A., Treacher D.F., Turner R.C. (1985). Homeostasis Model Assessment: Insulin Resistance and Beta-Cell Function from Fasting Plasma Glucose and Insulin Concentrations in Man. Diabetologia.

[27] Inker L.A., Eneanya N.D., Coresh J., Tighiouart H., Wang D., Sang Y., Crews D.C., Doria A., Estrella M.M., Froissart M. (2021). New Creatinine- and Cystatin C-Based Equations to Estimate GFR without Race. N. Engl. J. Med..

[28] American Diabetes Association Professional Practice Committee 2 (2022). Classification and Diagnosis of Diabetes: Standards of Medical Care in Diabetes-2022. Diabetes Care.

[29] Whelton P.K., Carey R.M., Aronow W.S., Casey D.E., Collins K.J., Dennison Himmelfarb C., DePalma S.M., Gidding S., Jamerson K.A., Jones D.W. (2018). 2017 ACC/AHA/AAPA/ABC/ACPM/AGS/APhA/ASH/ASPC/NMA/PCNA Guideline for the Prevention, Detection, Evaluation, and Management of High Blood Pressure in Adults: A Report of the American College of Cardiology/American Heart Association Task Force on Clinical Practice Guidelines. Hypertension.

[30] Mach F., Baigent C., Catapano A.L., Koskinas K.C., Casula M., Badimon L., Chapman M.J., De Backer G.G., Delgado V., Ference B.A. (2020). 2019 ESC/EAS Guidelines for the Management of Dyslipidaemias: Lipid Modification to Reduce Cardiovascular Risk. Eur. Heart J..

[31] Papathanasiou G., Georgoudis G., Papandreou M., Spyropoulos P., Georgakopoulos D., Kalfakakou V., Evangelou A. (2009). Reliability Measures of the Short International Physical Activity Questionnaire (IPAQ) in Greek Young Adults. Hell. J. Cardiol..

[32] Sember V., Meh K., Sorić M., Starc G., Rocha P., Jurak G. (2020). Validity and Reliability of International Physical Activity Questionnaires for Adults across EU Countries: Systematic Review and Meta Analysis. Int. J. Environ. Res. Public Health.

[33] Katsouyanni K., Rimm E.B., Gnardellis C., Trichopoulos D., Polychronopoulos E., Trichopoulou A. (1997). Reproducibility and Relative Validity of an Extensive Semi-Quantitative Food Frequency Questionnaire Using Dietary Records and Biochemical Markers among Greek Schoolteachers. Int. J. Epidemiol..

[34] Panagiotakos D.B., Pitsavos C., Stefanadis C. (2006). Dietary Patterns: A Mediterranean Diet Score and Its Relation to Clinical and Biological Markers of Cardiovascular Disease Risk. Nutr. Metab. Cardiovasc. Dis..

[35] Zaragoza-Martí A., Cabañero-Martínez M.J., Hurtado-Sánchez J.A., Laguna-Pérez A., Ferrer-Cascales R. (2018). Evaluation of Mediterranean Diet Adherence Scores: A Systematic Review. BMJ Open.

[36] Anastasiou C.A., Karfopoulou E., Yannakoulia M. (2015). Weight Regaining: From Statistics and Behaviors to Physiology and Metabolism. Metabolism.

[37] Paixão C., Dias C.M., Jorge R., Carraça E.V., Yannakoulia M., de Zwaan M., Soini S., Hill J.O., Teixeira P.J., Santos I. (2020). Successful Weight Loss Maintenance: A Systematic Review of Weight Control Registries. Obes. Rev..

[38] Yannakoulia M., Poulimeneas D., Mamalaki E., Anastasiou C.A. (2019). Dietary Modifications for Weight Loss and Weight Loss Maintenance. Metabolism.

[39] Zhang J., Wang H., Wang Z., Huang F., Zhang X., Du W., Su C., Ouyang Y., Li L., Bai J. (2021). Trajectories of Dietary Patterns and Their Associations with Overweight/Obesity among Chinese Adults: China Health and Nutrition Survey 1991–2018. Nutrients.

[40] Kang M., Boushey C.J., Shvetsov Y.B., Setiawan V.W., Paik H.-Y., Wilkens L.R., Le Marchand L., Park S.-Y. (2021). Changes in Diet Quality and Body Weight over 10 Years: The Multiethnic Cohort Study. Br. J. Nutr..

[41] Angulo E., Stern D., Castellanos-Gutiérrez A., Monge A., Lajous M., Bromage S., Fung T.T., Li Y., Bhupathiraju S.N., Deitchler M. (2021). Changes in the Global Diet Quality Score, Weight, and Waist Circumference in Mexican Women. J. Nutr..

[42] Panagiotakos D.B., Chrysohoou C., Pitsavos C., Stefanadis C. (2006). Association between the Prevalence of Obesity and Adherence to the Mediterranean Diet: The ATTICA Study. Nutrition.

[43] Georgoulis M., Damigou E., Chrysohoou C., Barkas F., Anastasiou G., Kravvariti E., Tsioufis C., Liberopoulos E., Sfikakis P.P., Pitsavos C. (2023). Mediterranean Diet Trajectories and 20-Year Incidence of Cardiovascular Disease: The ATTICA Cohort Study (2002–2022). Nutr. Metab. Cardiovasc. Dis..

[44] Lichtenstein A.H., Appel L.J., Vadiveloo M., Hu F.B., Kris-Etherton P.M., Rebholz C.M., Sacks F.M., Thorndike A.N., Van Horn L., Wylie-Rosett J. (2021). 2021 Dietary Guidance to Improve Cardiovascular Health: A Scientific Statement From the American Heart Association. Circulation.

[45] Lotfi K., Saneei P., Hajhashemy Z., Esmaillzadeh A. (2022). Adherence to the Mediterranean Diet, Five-Year Weight Change, and Risk of Overweight and Obesity: A Systematic Review and Dose-Response Meta-Analysis of Prospective Cohort Studies. Adv. Nutr..

[46] Twells L.K., Janssen I., Kuk J.L. Canadian Adult Obesity Clinical Practice Guidelines: Epidemiology of Adult Obesity. https://obesitycanada.ca/guidelines/epidemiology.

[47] Mancini J.G., Filion K.B., Atallah R., Eisenberg M.J. (2016). Systematic Review of the Mediterranean Diet for Long-Term Weight Loss. Am. J. Med..

[48] Bendall C.L., Mayr H.L., Opie R.S., Bes-Rastrollo M., Itsiopoulos C., Thomas C.J. (2018). Central Obesity and the Mediterranean Diet: A Systematic Review of Intervention Trials. Crit. Rev. Food Sci. Nutr..

[49] Barnard N.D., Alwarith J., Rembert E., Brandon L., Nguyen M., Goergen A., Horne T., do Nascimento G.F., Lakkadi K., Tura A. (2022). A Mediterranean Diet and Low-Fat Vegan Diet to Improve Body Weight and Cardiometabolic Risk Factors: A Randomized, Cross-over Trial. J. Am. Nutr. Assoc..

[50] Estruch R., Ros E., Salas-Salvadó J., Covas M.-I., Corella D., Arós F., Gómez-Gracia E., Ruiz-Gutiérrez V., Fiol M., Lapetra J. (2018). Primary Prevention of Cardiovascular Disease with a Mediterranean Diet Supplemented with Extra-Virgin Olive Oil or Nuts. N. Engl. J. Med..

[51] Konieczna J., Ruiz-Canela M., Galmes-Panades A.M., Abete I., Babio N., Fiol M., Martín-Sánchez V., Estruch R., Vidal J., Buil-Cosiales P. (2023). An Energy-Reduced Mediterranean Diet, Physical Activity, and Body Composition: An Interim Subgroup Analysis of the PREDIMED-Plus Randomized Clinical Trial. JAMA Netw. Open.

[52] Martínez-González M.A., Buil-Cosiales P., Corella D., Bulló M., Fitó M., Vioque J., Romaguera D., Martínez J.A., Wärnberg J., López-Miranda J. (2019). Cohort Profile: Design and Methods of the PREDIMED-Plus Randomized Trial. Int. J. Epidemiol..

[53] Serra-Majem L., Tomaino L., Dernini S., Berry E.M., Lairon D., Ngo de la Cruz J., Bach-Faig A., Donini L.M., Medina F.-X., Belahsen R. (2020). Updating the Mediterranean Diet Pyramid towards Sustainability: Focus on Environmental Concerns. Int. J. Environ. Res. Public Health.

[54] Bach-Faig A., Berry E.M., Lairon D., Reguant J., Trichopoulou A., Dernini S., Medina F.X., Battino M., Belahsen R., Miranda G. (2011). Mediterranean Diet Pyramid Today. Science and Cultural Updates. Public Health Nutr..

[55] Moussavi N., Gavino V., Receveur O. (2008). Could the Quality of Dietary Fat, and Not Just Its Quantity, Be Related to Risk of Obesity?. Obesity.

[56] Salleh S.N., Fairus A.A.H., Zahary M.N., Bhaskar Raj N., Mhd Jalil A.M. (2019). Unravelling the Effects of Soluble Dietary Fibre Supplementation on Energy Intake and Perceived Satiety in Healthy Adults: Evidence from Systematic Review and Meta-Analysis of Randomised-Controlled Trials. Foods.

[57] Merra G., Noce A., Marrone G., Cintoni M., Tarsitano M.G., Capacci A., De Lorenzo A. (2020). Influence of Mediterranean Diet on Human Gut Microbiota. Nutrients.

[58] Muscogiuri G., Cantone E., Cassarano S., Tuccinardi D., Barrea L., Savastano S., Colao A. (2019). Gut Microbiota: A New Path to Treat Obesity. Int. J. Obes. Suppl..

[59] Townsend J.R., Kirby T.O., Sapp P.A., Gonzalez A.M., Marshall T.M., Esposito R. (2023). Nutrient Synergy: Definition, Evidence, and Future Directions. Front. Nutr..

[60] Bes-Rastrollo M., Sánchez-Villegas A., de la Fuente C., de Irala J., Martinez J.A., Martínez-González M.A. (2006). Olive Oil Consumption and Weight Change: The SUN Prospective Cohort Study. Lipids.

[61] Zamora Zamora F., Martínez Galiano J.M., Gaforio Martínez J.J., Delgado Rodríguez M. (2018). [Olive Oil and Body Weight. Systematic Review and Meta-Analysis of Randomized Controlled Trials]. Rev. Esp. Salud Publica.

[62] Hill J.O. (2006). Understanding and Addressing the Epidemic of Obesity: An Energy Balance Perspective. Endocr. Rev..

[63] Mozaffarian D. (2017). Foods, Obesity, and Diabetes-Are All Calories Created Equal?. Nutr. Rev..

[64] Mozaffarian D. (2016). Dietary and Policy Priorities for Cardiovascular Disease, Diabetes, and Obesity: A Comprehensive Review. Circulation.

[65] Koutras Y., Chrysostomou S., Poulimeneas D., Yannakoulia M. (2022). Examining the Associations between a Posteriori Dietary Patterns and Obesity Indexes: Systematic Review of Observational Studies. Nutr. Health.

[66] Blundell J.E., Dulloo A.G., Salvador J., Frühbeck G., on behalf of the EASO SAB Working Group on BMI (2014). Beyond BMI—Phenotyping the Obesities. Obes. Facts.

